# Multiparametric Magnetic Resonance Investigation of Brain Adaptations to 6 Days at 4350 m

**DOI:** 10.3389/fphys.2016.00393

**Published:** 2016-09-08

**Authors:** Samuel Verges, Thomas Rupp, Marjorie Villien, Laurent Lamalle, Irène Troprés, Camille Poquet, Jan M. Warnking, François Estève, Pierre Bouzat, Alexandre Krainik

**Affiliations:** ^1^HP2 Laboratory, Université Grenoble AlpesGrenoble, France; ^2^U1042, Institut National de la Santé et de la Recherche MédicaleGrenoble, France; ^3^Inter-Universitary Laboratory of Human Movement Biology, Université Savoie Mont BlancChambéry, France; ^4^Grenoble Institute of Neurosciences, Université Grenoble AlpesGrenoble, France; ^5^SFR1, Université Grenoble AlpesGrenoble, France; ^6^U836, Institut National de la Santé et de la Recherche MédicaleGrenoble, France

**Keywords:** hypoxia, altitude, brain volume, diffusion, cerebral blood flow, intracranial pressure

## Abstract

**Objective:** Hypoxic exposure in healthy subjects can induce acute mountain sickness including headache, lethargy, cerebral dysfunction, and substantial cerebral structural alterations which, in worst case, can lead to potentially fatal high altitude cerebral edema. Within this context, the relationships between high altitude-induced cerebral edema, changes in cerebral perfusion, increased brain parenchyma volume, increased intracranial pressure, and symptoms remain unclear.

**Methods:** In 11 subjects before and after 6 days at 4350 m, we performed multiparametric magnetic resonance investigations including anatomical, apparent diffusion coefficient and arterial spin labeling sequences.

**Results:** After the altitude stay, while subjects were asymptomatic, white matter volume (+0.7 ± 0.4%, *p* = 0.005), diffusion (+1.7 ± 1.4%, *p* = 0.002), and cerebral blood flow (+28 ± 38%; *p* = 0.036) were significantly increased while cerebrospinal fluid volume was reduced (−1.4 ± 1.1%, *p* = 0.009). Optic nerve sheath diameter (used as an index of increased intracranial pressure) was unchanged from before (5.84 ± 0.53 mm) to after (5.92 ± 0.60 mm, *p* = 0.390) altitude exposure. Correlations were observed between increases in white matter volume and diffusion (rho = 0.81, *p* = 0.016) and between changes in CSF volume and changes in ONSD s (rho = −0.92, *p* = 0.006) and symptoms during the altitude stay (rho = −0.67, *p* = 0.031).

**Conclusions:** These data demonstrate white matter alterations after several days at high altitude when subjects are asymptomatic that may represent the normal brain response to prolonged high altitude exposure.

## Introduction

Over the past decade, several studies reported that acute normobaric hypoxic exposure (inspiratory O_2_ fraction, FiO_2_ = 0.11–0.12) lasting for less than 1 h (Dubowitz et al., 2009; Rupp et al., 2014) up to 22 h (Bailey et al., 2006; Kallenberg et al., 2007; Mairer et al., 2012; Lawley et al., 2013, 2014) increases brain gray (GM) and/or white (WM) matter volumes assessed by magnetic resonance imaging. The underlying mechanisms and the consequences of increased brain parenchyma volume during acute hypoxic exposure remain unclear. It might be associated with cerebral subedema [as suggested by changes in apparent diffusion coefficient (ADC) and fractional anisotropy for instance (Kallenberg et al., 2007; Schoonman et al., 2008; Mairer et al., 2012; Lawley et al., 2013; Rupp et al., 2014)] or with increased cerebral blood volume (Dubowitz et al., 2009; Wilson et al., 2009, 2013; Lawley et al., 2014), while its role regarding symptoms of acute mountain sickness (AMS: headache, dizziness, fatigue, nausea, etc) is debated. More specifically, reduced ADC during acute hypoxic exposure might be associated with AMS symptoms while increased ADC and reduced fractional anisotropy might be part of the normal physiological response to hypoxia (Kallenberg et al., 2007; Schoonman et al., 2008; Hunt et al., 2013; Lawley et al., 2013; Rupp et al., 2014). Increased brain parenchyma volume may be important regarding symptoms such as headache by promoting increased intracranial pressure (Bailey et al., 2006; Kallenberg et al., 2007; Lawley et al., 2014).

Sagoo et al. (2016) recently exposed 12 subjects to a FiO_2_ = 0.12 for 22 h with serial magnetic resonance imaging sequences. Together with symptoms of AMS, subjects showed significant increases in GM and WM volumes and ADC and a significant reduction in intracranial cerebrospinal fluid (CSF). The increase in WM volume only correlated with symptoms of AMS. These authors suggested that the development of cerebral subedema with increased brain parenchyma volumes as well as venous outflow restriction may induce an increase in intracranial pressure probably responsible for symptoms Lawley et al. (2014) explored the relationship between changes in brain volume, magnetic resonance imaging-derived intracranial pressure, and AMS symptoms in 13 subjects exposed to FiO_2_ = 0.12 for 10 h. While total brain volume significantly increased and CSF volume decreased for the whole group after hypoxic exposure, subjects with AMS symptoms had a significant increase in intracranial pressure compared to subjects without AMS. These data support the notion that increased intracranial pressure might be a key mechanism underlying AMS symptoms.

Optic nerve sheath diameter (ONSD) was recently studied in healthy subjects exposed to high altitude (Fagenholz et al., 2009; Strapazzon et al., 2014) since this parameter was correlated with intracranial pressure value in critically ill patients (Geeraerts et al., 2008). An enlargement of ONSD during high altitude exposure has been reported and may be associated with altitude-induced increase in intracranial pressure and symptoms (Fagenholz et al., 2009; Strapazzon et al., 2014). While ONSD was measured at high altitude by ultrasonography, magnetic resonance imaging has been used to validate ONSD as an index of increased intracranial pressure (Geeraerts et al., 2008; Steinborn et al., 2011). Hence, assessing ONSD together with other magnetic resonance imaging measurements of brain parenchyma volume and subedema and cerebral perfusion should provide useful information regarding the mechanisms of brain responses to high altitude and the putative relationships between cerebral subedema, changes in cerebral perfusion, increased brain parenchyma volume, increased intracranial pressure, and AMS symptoms.

In the present study, we aimed to assess changes in brain volume, ADC, and ONSD following several days of high altitude exposure, when symptoms of AMS have disappeared (as opposed to previous studies having investigated cerebral subedema and changes in brain volume within the first 24 h) but when increased cerebral blood flow is still present (Villien et al., 2013). We hypothesized that WM volume would be increased together with increased ADC and reduced fractional anisotropy but unchanged ONSD (since we expected intracranial pressure and AMS symptoms to be normalized after 6 days at altitude), which would characterize the normal brain response to prolonged hypobaric hypoxic exposure.

## Subjects and methods

### Subjects

Eleven healthy male subjects (28 ± 8 years old) were recruited to participate to this study and provided written informed consent. Participants were recreational climbers, taking no medication, and having no history of cardiovascular, cerebrovascular and respiratory diseases. Their altitude of residence was < 1000 m (200–900 m), they were not acclimatized to high altitude (no night above 1500 m or sojourn above 2500 m of altitude over the past 3 months) and received no treatment to prevent acute mountain sickness. The study was approved by the local ethics committee and performed according to the Declaration of Helsinki (registration number: RCB2011-A00071-40, ClinicalTrials.gov ID: NCT01565603).

### Experimental design

Magnetic resonance imaging examinations including volumetric, DWI, and arterial spin labeling sequences were performed in Grenoble (212 m) before and within 6 h after returning from 6 days at 4350 m of altitude (Observatoire Vallot, Mont Blanc, Chamonix, France). Subjects underwent helicopter transport lasting for 10 min between the valley and high altitude (4350 m). They remained for 6 days in the Observatoire Vallot, without further ascent, and without performing any climbing or prolonged physical activities. They kept their usual diet and slept for at least 7 h per night. Because 11 subjects could not be evaluated simultaneously during and immediately after the altitude stay, the experiment was performed over 2 weeks, a subgroup of 5 (or 6) subjects being exposed to high altitude and investigated according to the same protocol during each week. Data regarding arterial spin labeling have been previously reported (Villien et al., 2013).

### Magnetic resonance imaging examination

Before and after the altitude stay, magnetic resonance images were acquired using a whole body scanner at 3 Tesla (Philips Achieva 3.0T TX, Best, Netherlands) equipped with a 32-channel head receive array. The following sequences were applied:

(i) High resolution 3D T_1_-weighted MP-RAGE gradient-echo sequence covering the whole brain: 800 ms inversion time, linear order phase encoding, TFE factor of 55 with 2300 ms segment repetition time, 4 ms echo time, 25 ms repetition time, 15° flip angle, sagittal orientation with 256 mm (H-F) × 241 mm (A-P) × 160 mm (L-R) field of view, 272 × 234 × 100 nominal acquisition matrix, SENSE acceleration factors of 2.2 in A-P and 1.6 in L-R phase encoding directions, reconstructed to 0.89 × 0.89 × 1.20 mm resolution;
(ii) High resolution 3D T_2_-weighted TSE sequence (BrainView T2) covering the whole brain: linear order phase encoding, half-Fourier factor of 0.8, TSE factor of 168, 233 ms echo time, 2500 ms repetition time, 90° flip angle, axial orientation with 256 mm (A-P) × 224 mm (L-R) × 224 mm (H-F) field of view, SENSE acceleration factors of 1.2 in L-R and F-H phase encoding directions, 256 × 223 × 224 acquisition matrix reconstructed to 1.00 mm isotropic resolution.
(iii) Diffusion-weighted spin-echo single-shot echoplanar imaging: 59 axial slices acquired in interleaved order and distributed over two packages, 2.25 mm slice-thickness, 0.25 mm inter-slice gap, 220 mm square field of view, 88 × 85 nominal acquisition matrix, SENSE acceleration factor of 3 in L-R echoplanar imaging blip gradient direction, 96 × 96 reconstruction to 2.29 mm in plane resolution, 74 ms echo time, 2939 ms repetition time between packages, 90° flip angle, diffusion weighting b factor of 1000 s/mm^2^ applied in 6 directions. A standard gradient echo data set at two echo times was also acquired in view of B_0_ field map estimation for magnetic field susceptibility-related geometric distortion correction;
(iv) Pseudo-continuous arterial spin labeling acquisition, as previously described (Villien et al., 2013): WET pre-saturation, 1650 ms label, 1525 ms post-label delay, multi-slice single-shot EPI readout (3 × 3 × 6 mm^3^, 20 slices, 12 ms echo time, sense-factor 2.5), repetition time of 4 s.

Standard processing of the anatomical images was performed using SPM8 (Wellcome Department of Cognitive Neurology, London, UK) and FSL 4.1.9 (Analysis Group, FMRIB, Oxford, UK) softwares as described previously (Rupp et al., 2014). All individual images were spatially normalized to images recorded before altitude exposure. Segmentation of T_1_ images was performed to obtain GM and WM volumes while segmentation of T_2_ images was performed to obtain CSF volume. Data from one subject had to be excluded due to excessive head motion.

ADC and fractional anisotropy maps were computed from the eigenvalues of the diffusion tensor as estimated by FSL, after correction for eddy currents and affine motion (FSL's “eddy_correct” tool) and masking (mask extracted from *b* = 0 image using FSL's “bet” tool). These maps were then coregistered with the corresponding T1 images. WM and GM masks obtained from segmentation of T1 images were used to compute individual WM or GM ADC and fractional anisotropy maps.

Arterial spin labeling data were analyzed as previously described (Villien et al., 2013) and changes in cerebral blood flow from before to after altitude were converted into changes in cerebral blood volume based on the 0.38 power-law relationship (Grubb et al., 1974; Dubowitz et al., 2009). According to this relationship, changes in cerebral blood volume can be estimated from changes in cerebral blood flow based on the following equation:

Cerebral blood volume = 0.80 × Cerebral blood flow^0.38^

**Figure 1:**
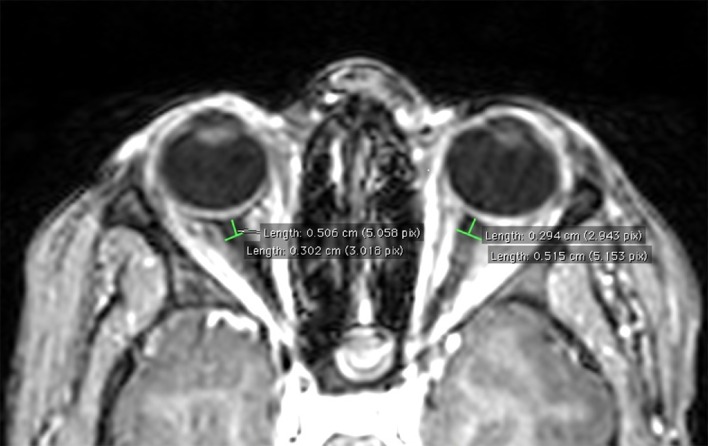
**Example of ONSD measurement on T1-weighted magnetic resonance image in a representative subject**.

ONSD was assessed according to Geeraerts et al. (2008). Briefly, T1-weighted magnetic resonance imaging was used to measure ONSD in an axis perpendicular to the optic nerve, 3 mm behind the globe using an electronic caliper (Figure 1). Three consecutive measurements were done bilaterally by the same experimenter (PC) blinded for experimental conditions (before-after altitude). ONSD was not available in three subjects due to excessive head motion and technical issue.

### Symptoms and cardiorespiratory parameters

Every morning at high altitude, subjects were asked to complete self-reported questionnaires for AMS evaluation according to the Lake Louise score (five items) (Roach et al., 1993) and to score perceived headache on a 10-cm visual analog scale. Cumulative Lake Louise score and perceived headache scores were obtained by adding each individual morning score over the 6 days at high altitude. Before each magnetic resonance imaging examination, the following cardiorespiratory parameters were assessed: heart rate and non-invasive blood pressure (Dinamap, GE Medical Systems Inc., Milwaukee, WI), arterial oxygen saturation using finger-pulse oximetry and end-tidal CO_2_ partial pressure (Maglife, Schiller AG, Baan, Switzerland).

### Statistical analysis

All statistical procedures were completed on Statistica version 10 (Statsoft, Tulsa, OK, USA). The Wilcoxon test was used to compare values before and after altitude exposure. Relationships between magnetic resonance imaging parameters were evaluated by Spearman correlation coefficient (rho). An alpha level of 0.05 was used as the cut-off for significance. All data are presented as mean values ± SD.

## Results

### Symptoms and cardiorespiratory parameters

Lake Louise score and perceived headache increased during the first days at high altitude with individual maximum scores reached within the first 3 days (mean maximal Lake Louise score 6 ± 3 pts (range of individual values: 2–10 pts), mean maximal perceived headache 57 ± 16 mm (range of individual values: 32–76 mm); all *p* < 0.05 compared to sea level) while on the last day at high altitude (Day 6) scores were not significantly different anymore compared to sea level (Lake Louise score 1 ± 1 pts (range of individual values: 0–3 pts), headache 13 ± 15 mm (range of individual values: 0–53 mm); all *p* > 0.05).

Heart rate (61 ± 8 bpm before altitude vs. 63 ± 8 bpm after altitude, *p* = 0.152), mean arterial blood pressure (104 ± 6 vs. 106 ± 8 mmHg, *p* = 0.303) and arterial oxygen saturation (97 ± 1 vs. 98 ± 1%, *p* = 0.198) did not differ before and after altitude exposure while end-tidal CO_2_ partial pressure was significantly lower after altitude exposure (41 ± 5 vs. 33 ± 4 mmHg, *p* < 0.001).

### Cerebral volumes

**Figure 2:**
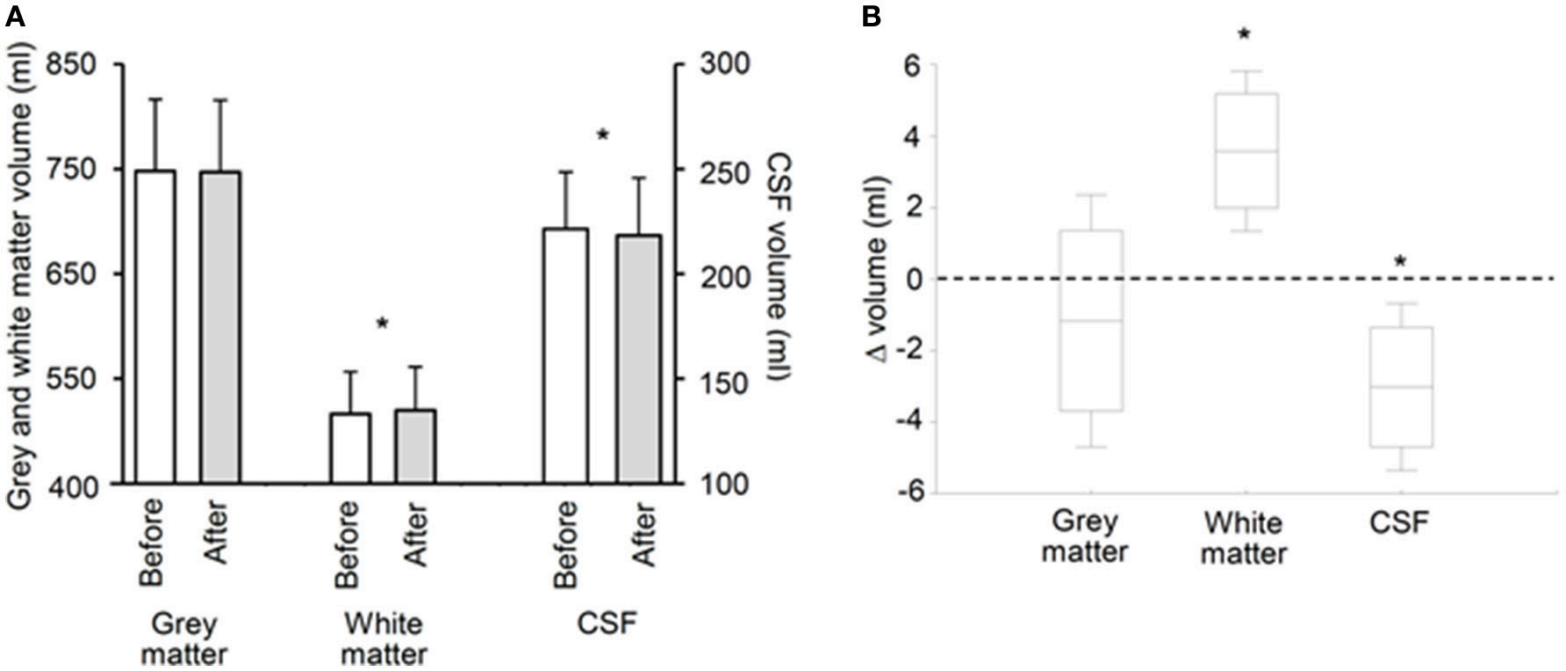
**Gray and white matter and cerebrospinal fluid (CSF) volumes before and after 6 days at 4350 m [(A), absolute values; (B), before-after changes]**. Data points are means ± SD, boxes are 95% confidence intervals. ^*^Significant difference between before and after (*p* < 0.01).

**Figure 3:**
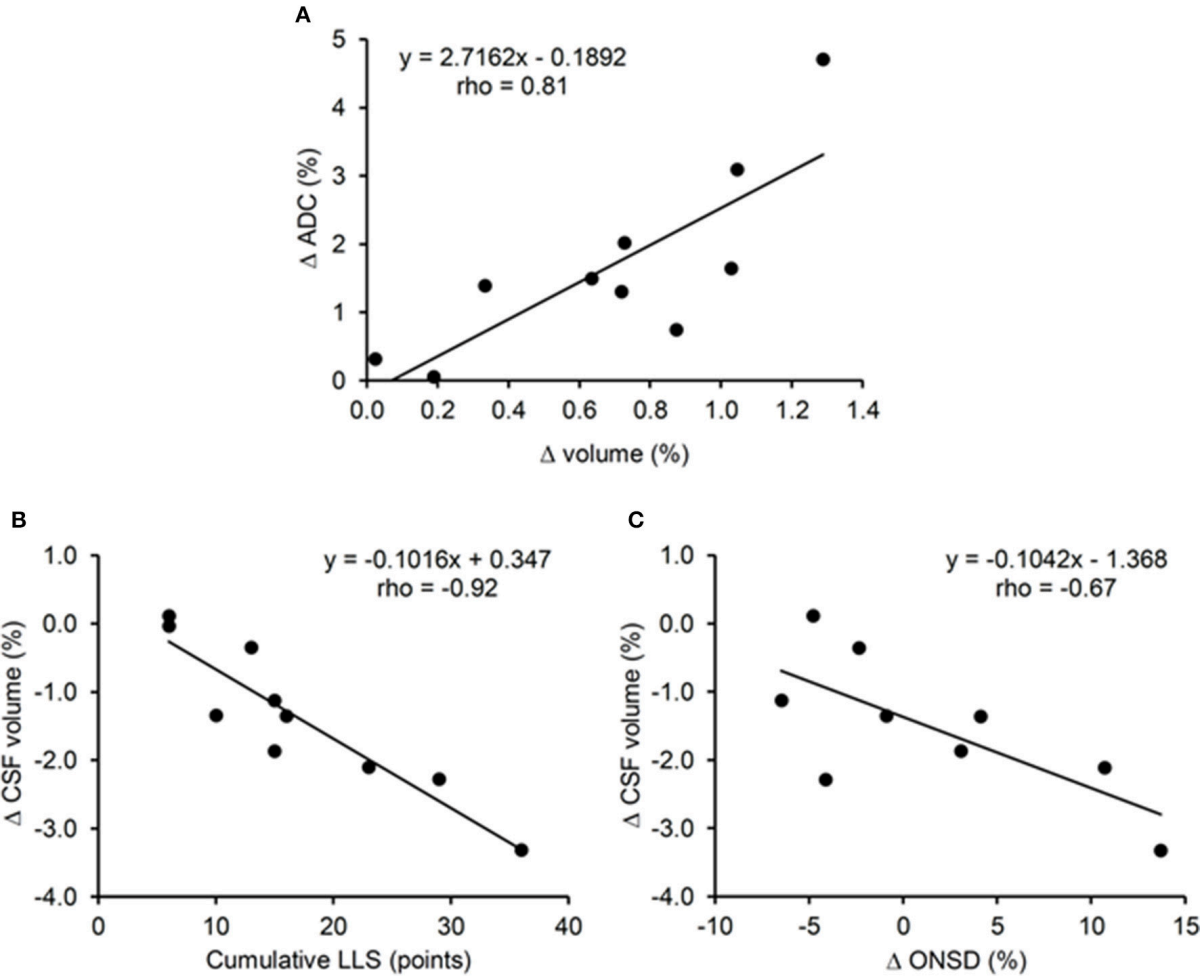
**Correlations between changes in white matter apparent diffusion coefficient (ADC) and volume (A), changes in cerebrospinal fluid (CSF) volume and cumulative Lake Louise Score (LLS) (B), changes in CSF volume and changes on optic nerve sheath diameter (ONSD) (C) during 6 days at 4350 m**.

A significant increase in WM volume (+0.7 ± 0.4%, *p* = 0.005) was observed after altitude exposure compared to before while no significant change were observed for GM volume (Figure 2). Total brain parenchyma volume (WM+GM) did not change significantly (1265 ± 106 ml before altitude vs. 1267 ± 107 ml after altitude, *p* = 0.441). CSF volume was significantly reduced after altitude exposure (−1.4 ± 1.1%, *p* = 0.009; Figure 2). Changes in CSF volume only correlated with cumulative Lake Louise score (Figure 3B) and perceived headache (rho = −0.93, *p* < 0.001).

### Diffusion

**Figure 4:**
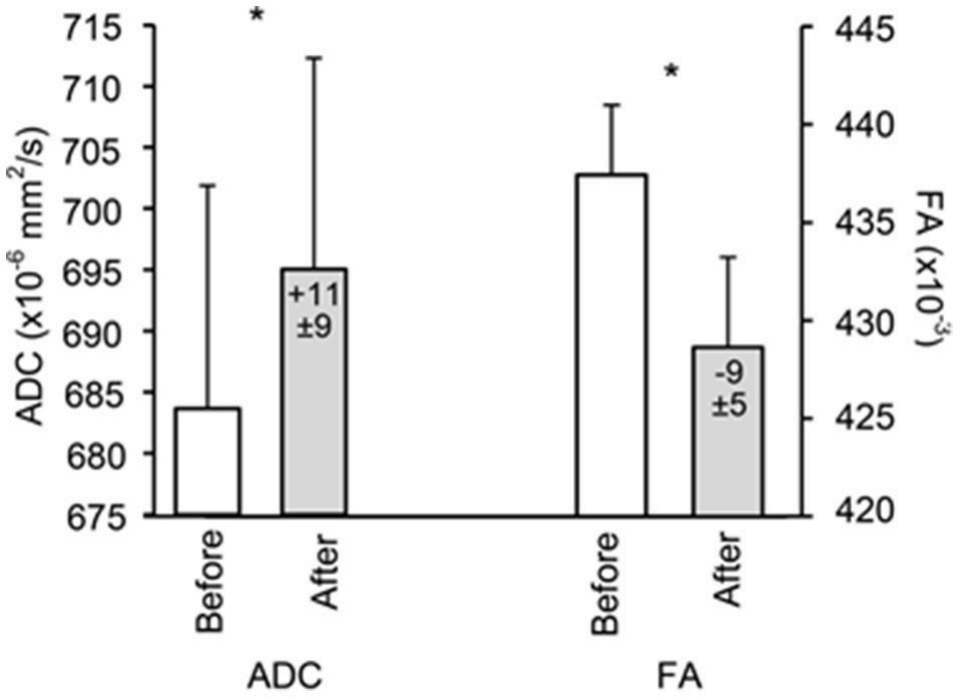
**White matter apparent diffusion coefficient (ADC) and fractional anisotropy (FA) before and after 6 days at 4350 m**. Values in the bars indicate difference compared to before altitude. Data points are means ± SD. ^*^Significant difference between before and after (*p* < 0.01).

WM ADC was significantly increased after altitude exposure (Figure 4) while no change in GM ADC was observed (854 ± 33 × 10^−6^ mm^2^/s before altitude vs. 850 ± 33 × 10^−6^ mm^2^/s after altitude, *p* = 0.508). Similarly, WM fractional anisotropy was significantly reduced after altitude exposure (Figure 4) while GM fractional anisotropy did not change (208 ± 133 vs. 205 ± 130 × 10^−3^, *p* = 0.355). The increases in WM volume and WM ADC were significantly correlated (Figure 3A).

### Cerebral blood flow and volume

WM (33.1 ± 8.3 ml·100 g^−1^·min^−1^ before altitude vs. 40.1 ± 5.1 ml·100 g^−1^·min^−1^ after altitude; *p* = 0.036) and GM (72.5 ± 14.8 vs. 82.1 ± 10.0 ml·100 g^−1^·min^−1^; *p* = 0.047) cerebral blood flow significantly increased after altitude exposure. Neither changes in cerebral blood flow nor in cerebral blood volume (WM +2.5 ± 3.4 ml, GM +3.4 ± 5.0 ml) after altitude correlated with changes in parenchyma volume, ADC and fractional anisotropy (all *p* > 0.05).

### ONSD

**Figure 5:**
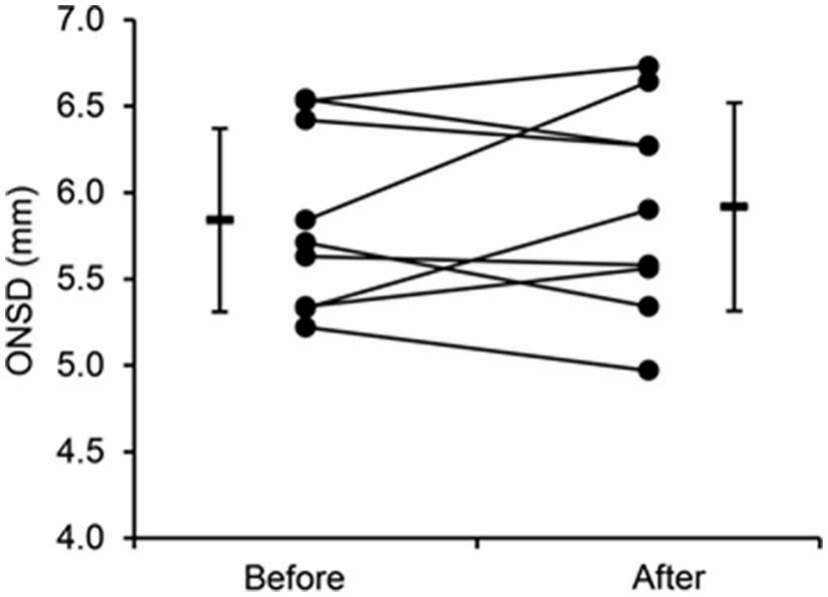
**Individual and mean values of optic nerve sheath diameter (ONSD) before and after 6 days at 4350 m**.

ONSD was not significantly different before and after altitude exposure (5.84 ± 0.53 mm before altitude vs. 5.92 ± 0.60 mm after altitude, *p* = 0.390; Figure 5), with an average increase of 1.4 ± 0.1% after altitude exposure. Changes in ONSD were negatively correlated with changes in CSF volume only (Figure 3C). Although no significant correlation was observed between changes in ONSD after altitude exposure and symptoms during the altitude stay (correlation between ONSD changes and cumulative Lake Louise score, rho = 0.62, *p* = 0.08; correlation between ONSD changes and cumulative perceived headache, rho = 0.66, *p* = 0.06), the three subjects with the largest ONSD increase from before to after altitude exposure (individual ONSD increases: +4.1, +10.7, +13.7%) were those reporting the largest perceived headache during altitude exposure (individual perceived headache: 72, 66, 76 mm). The mean intra-observer coefficient of variation for repeated measures of ONSD before altitude exposure was 2.5%.

## Discussion

The present results suggest that prolonged high altitude exposure induces brain parenchyma changes specifically in WM characterized by significant increases in volume and ADC, fractional anisotropy reduction, while cerebral blood flow was enhanced in both WM and GM. Concomitantly, CSF volume was significantly reduced while unchanged ONSD did not support a significant increase in intracranial pressure at the group level. These data demonstrate for the first time that cerebral morphometric changes previously reported after few hours of hypoxia are still present after a prolonged altitude exposure when subjects become acclimatized and do no report AMS symptoms anymore.

The significant increase in WM volume observed in all subjects of the present study is in accordance with previous results from our laboratory following shorter (11 h) hypoxic exposure as well as with previous results from the literature (Hackett et al., 1998; Schoonman et al., 2008; Zhang et al., 2012). Conversely, other results report changes in volume or diffusion in the GM only (Morocz et al., 2001; Lawley et al., 2014). The reason for these discrepancies is unclear but WM may be more sensitive to vasogenic edema because of smaller density and therefore less resistance to invasion by fluid (Fishman, 1975; Klatzo, 1987; Hackett et al., 1998). The concomitant increase in WM volume and ADC and the correlation between both changes (Figure 3A) suggest that an increase in WM extracellular water may be associated with the increased WM volume. The reduction in WM fractional anisotropy further supports increased WM extracellular water after altitude exposure.

Previous studies having investigated climbers before and several days/weeks after a prolonged (several weeks) stay at high altitude (>5000 m) reported reduced WM or GM volume/density (Di Paola et al., 2008; Foster et al., 2015; Kottke et al., 2015). Zhang et al. (2013) also reported changes in regional GM volume (decreased GM volume in the right postcentral gyrus and right superior temporal gyrus; increased GM volume in the right middle frontal gyrus, right parahippocampal gyrus, right inferior, and middle temporal gyri, bilateral inferior ventral pons and right cerebellum crus 1) in soldiers who stayed for 2 years at high altitude (2300–4400 m). These contrasting results compared to the present study regarding individuals staying for prolonged duration at high altitude can be explained by differences in the level and duration of high altitude exposure, by the time delay between the end of the altitude exposure and the magnetic resonance imaging examinations and by potential changes in physical activity and nutritional behaviors at high altitude.

The cerebral blood flow increase observed in the WM could favor extravascular fluid shift and consequently participate to the WM ADC and volume increase. There was however no correlation between changes in cerebral blood flow and changes in ADC or cerebral volume. In addition, cerebral blood flow also increased in GM without concomitant changes in GM volume and ADC. An increase in venous blood volume is an additional mechanism that has been recently discussed to explain increased cerebral volume, enhanced intracranial pressure, and finally symptoms following prolonged hypoxic exposure (Wilson et al., 2013; Lawley et al., 2014). An increase in venous blood volume due to impaired venous blood drainage would enhance cerebral blood pressure and due to the cerebral autoregulation, induce cerebral vasoconstriction. In the present study, the enhanced cerebral blood flow rather suggests vasodilation. In addition, no relationship between changes in cerebral blood volume calculated from changes in cerebral blood flow and cerebral volume was observed. Although these results do not support a potential role of venous volume regarding changes in brain volume and intracranial pressure as recently suggested (Wilson et al., 2013; Lawley et al., 2014), specific measurements of cerebral venous volume and blood pressure would be needed to assess this potent mechanism.

An important specificity of the present study is that magnetic resonance imaging measurements were performed after 6 days at high altitude when symptoms of AMS disappeared as opposed to previous studies of cerebral changes within the first 24 h of hypoxic exposure. The persistence of increased WM volume and ADC and reduced fractional anisotropy indicates these cerebral changes are independent of AMS and may represent normal responses to high altitude as previously observed in asymptomatic subjects (Kallenberg et al., 2007; Hunt et al., 2013). Alternatively, it may be suggested that these WM volume and diffusion changes represent mechanisms responsible for symptoms during the first hours at high altitude which persist but have been compensated after several days at high altitude. Hence, since no significant change in total brain volume was observed, it can be suggested that the increase in WM volume was buffered by the reduction in CSF volume (Figure 2). The correlation between changes in CSF volume and cumulative Lake Louise score (Figure 3B) may suggest that the later mechanism was particularly required in subjects with the greatest symptoms. As a consequence, intracranial pressure might be unchanged after 6 days of high altitude exposure, explaining the absence of AMS symptoms including headache, as opposed to the first hours at altitude where increased parenchyma volume may be associated with increased intracranial pressure and symptoms (Lawley et al., 2014).

To evaluate potential changes in intracranial pressure during the altitude sojourn, we measured ONSD from anatomical magnetic resonance images as this parameter was shown to detect increased intracranial pressure in pathological or experimental conditions (Geeraerts et al., 2008; Steinborn et al., 2011). ONSD can also be measured using ultrasonography and several reports described an increase in ONSD measured by ultrasonography during high altitude exposure (Fagenholz et al., 2009; Strapazzon et al., 2014). Strapazzon et al. (2014) measured ONSD in healthy lowlanders before and over 8 days at 3830 m. ONSD increased within the first 24 h and remained enlarged until 8 days of altitude exposure. ONSD was also significantly correlated with Lake Louise score after 24 h of altitude exposure, i.e., the more symptomatic subjects had the largest ONSD. In the present study, no significant change in ONSD was observed after 6 days at 4350 m, suggesting no significant increase in intracranial pressure at the group level. The correlation between changes in CSF volume and changes in ONSD (Figure 3C) may suggest that subjects with the largest increase in intracranial pressure were those where large reduction in CSF volume were required to buffer the brain parenchyma volume increase and consequently the enhanced intracranial pressure. Although a tendency for more severe symptoms in subjects with the largest increase in ONSD was observed, no significant correlation between symptoms during the altitude sojourn, and changes in ONSD was observed. Nevertheless, the subjects who reported the most severe symptoms at the beginning of the altitude stay had the greatest increase in ONSD. Hence, we cannot exclude that an increase in ONSD which remains after several day of altitude exposure as observed in some subjects in the present study and in the study by Strapazzon et al. (2014) might reflect a significant increase in intracranial pressure within the first hours at high altitude which led to symptoms such as headache. Although intracranial hypertension and symptoms would have resorbed after several days at high altitude, ONSD would remain elevated as a consequence of the transient increase in intracranial pressure at the beginning of the altitude exposure. More studies are required to determine the pathophysiological significance and clinical relevance of ONSD measurements in the context of high altitude exposure (Lochner et al., 2015).

Several limitations of the present study should be acknowledged. The main limitation of the present study and most previous studies of brain alterations at altitude is the small sample size. By using helicopter transportation and a 6-day sojourn within a relatively comfortable location (Observatoire Vallot, Mont Blanc, Chamonix, France) and without prolonged physical effort, the present study minimized several confounding factors associated with high altitude exposure, and therefore increased the ability to detect high-altitude hypoxia-induced cerebral changes. While most previous magnetic resonance imaging studies investigated cerebral changes induced by normobaric hypoxia (i.e., by reducing FiO_2_ at sea level), the present study assessed the effect of hypobaric hypoxia. Although the present data have the advantage to be directly applicable to high altitude exposure, comparisons with previous data collected in normobaric hypoxia may be influenced by potential differences between normobaric and hypobaric hypoxic responses (Coppel et al., 2015; Ribon et al., 2016). Magnetic resonance imaging measurements after altitude exposure were performed in normoxia at sea level, i.e., while subjects recovered normal arterial oxygen saturation. Since the time delay between the end of high altitude exposure and the magnetic resonance imaging measurements was less than 6 h, we believed that most cerebral changes induced by high-altitude hypoxia persisted when magnetic resonance imaging investigations were conducted, although further studies are needed to clarify the recovery of high-altitude-induced cerebral changes. Also, compared to before altitude exposure, subjects were hypocapnic after altitude exposure due to altitude-induced hyperventilation. After several days at altitude however, the initial increase in cerebrospinal pH is thought to be partly compensated (Brugniaux et al., 2007) and it can therefore be expected that cerebral changes observed in the present study after altitude exposure were independent of cerebrospinal pH changes. Finally, measurements of regional cerebral morphometric changes are required to define regions potentially more susceptible to hypoxic exposure.

By investigating cerebral changes with multiparametric magnetic resonance imaging after several days of high altitude exposure, the present study demonstrated significant cerebral morphometric changes in subjects not reporting AMS symptoms anymore. Hence, increased WM volume and diffusion (indicating vasogenic subedema) as well as reduced CSF volume and unchanged ONSD (suggesting no intracranial hypertension) may represent the normal brain response to high-altitude hypoxia. AMS symptoms reported over the first hours at high altitude may nevertheless be induced by some of these mechanisms which might be compensated later on with acclimatization. Further multiparametric magnetic resonance imaging investigations are required to clarify the kinetic of brain structural alterations and interindividual differences regarding symptoms during acute and prolonged high altitude exposure.

## Author contributions

SV, TR, MV, LL, JW, FE, PB, and AK contributed to the conception and design of the work; SV, TR, MV, LL, IT, CP, JW, FE, PB, and AK contributed to data acquisition, analysis and interpretation; SV, TR, MV, LL, IT, CP, JW, FE, PB, and AK drafted the work and revised it critically; SV, TR, MV, LL, IT, CP, JW, FE, PB, and AK approved the final version to be published; SV, TR, MV, LL, IT, CP, JW, FE, PB, and AK agree to be accountable for all aspects of the work and ensure that questions related to the accuracy and integrity of all parts of the work are appropriately investigated and resolved.

### Conflict of interest statement

The authors declare that the research was conducted in the absence of any commercial or financial relationships that could be construed as a potential conflict of interest.
